# A Clinical Perspective on Herpetiform Aphthous Stomatitis: A Case Report on the Findings of 126 Oral Ulcers in a 21-Year-Old Male

**DOI:** 10.7759/cureus.114088

**Published:** 2026-08-06

**Authors:** Ramachandra Reddy Gowda Venkatesha, Vinitha Ganesan, Karthik Rajaram Mohan, Stanly Manohar Royan, Sindhuja Rajalingam

**Affiliations:** 1 Oral Medicine and Radiology, Vinayaka Mission's Sankarachariyar Dental College, Vinayaka Mission's Research Foundation, Salem, IND

**Keywords:** corticosteroids, gingiva, herpetiform aphthous ulcers, oral ulcer, tongue ulcer

## Abstract

Herpetiform aphthous ulcer (HAU), a rare clinical variant of aphthous ulcer, presents in the oral cavity as multiple, grouped tiny ulcers. These ulcers occur in the mobile, non-keratinized labial mucosa, soft palate, buccal mucosa, and ventral surface of the tongue and are known for their severe and debilitating pain. This article presents a unique and intriguing case of herpetiform aphthous ulcers in a 21-year-old male who reported severe soreness and burning sensations during the consumption of food. The intraoral clinical findings of this rare case of herpetiform aphthous ulcers revealed a total of 126 tiny oral ulcers, each measuring about 1 mm in diameter. Among these 126 tiny oral ulcers, 45 were located on the ventral surface of the tongue, 10 on the soft palate, 35 each on the right and left buccal mucosa and one on the maxillary labial mucosa. Treatment modalities are detailed in this case report.

## Introduction

The term 'aphthous' is derived from the Greek word *aphtha*, which means ulceration, to inflame, or set on fire [1]. Herpetiform aphthous ulcers (HAU) are a less common subtype of recurrent aphthous ulcers (RAU), characterized by multiple small, painful ulcers that can coalesce into larger lesions [1]. Despite their name, they are not caused by a viral infection and are often misdiagnosed due to their clinical resemblance to herpetic lesions [1,2]. Herpetiform aphthous ulcers comprise less than 5% of aphthous stomatitis cases [2]. They present as multiple, small ulcers (1 mm to 2 mm) that can merge to form larger, irregular-shaped ulcers, typically healing within two to four weeks without scarring [1,2]. The exact cause of HAU is unknown. Herpetiform aphthous ulcers share histologic and immunologic features with other types of RAU, suggesting a possible common underlying etiological mechanism. Factors such as local trauma, stress, systemic diseases, and nutritional deficiencies such as iron and vitamin B complex contribute to the development of HAU [1-3].

Herpetiform-type recurrent aphthous stomatitis (HRAS) or HAU are non-contagious, non-viral infections. They are a distinct form of recurrent aphthous stomatitis characterized by the presence of many small-sized ulcerations (1 mm to 3 mm in diameter) on the mobile non-keratinized mucosa, including the maxillary and mandibular labial mucosa, buccal mucosa, mucosa of the ventral surface of the tongue, and floor of the mouth. Typically, HAU lesions recover within seven to 10 days or in a month [4-6].

## Case presentation

**Figure 1:**
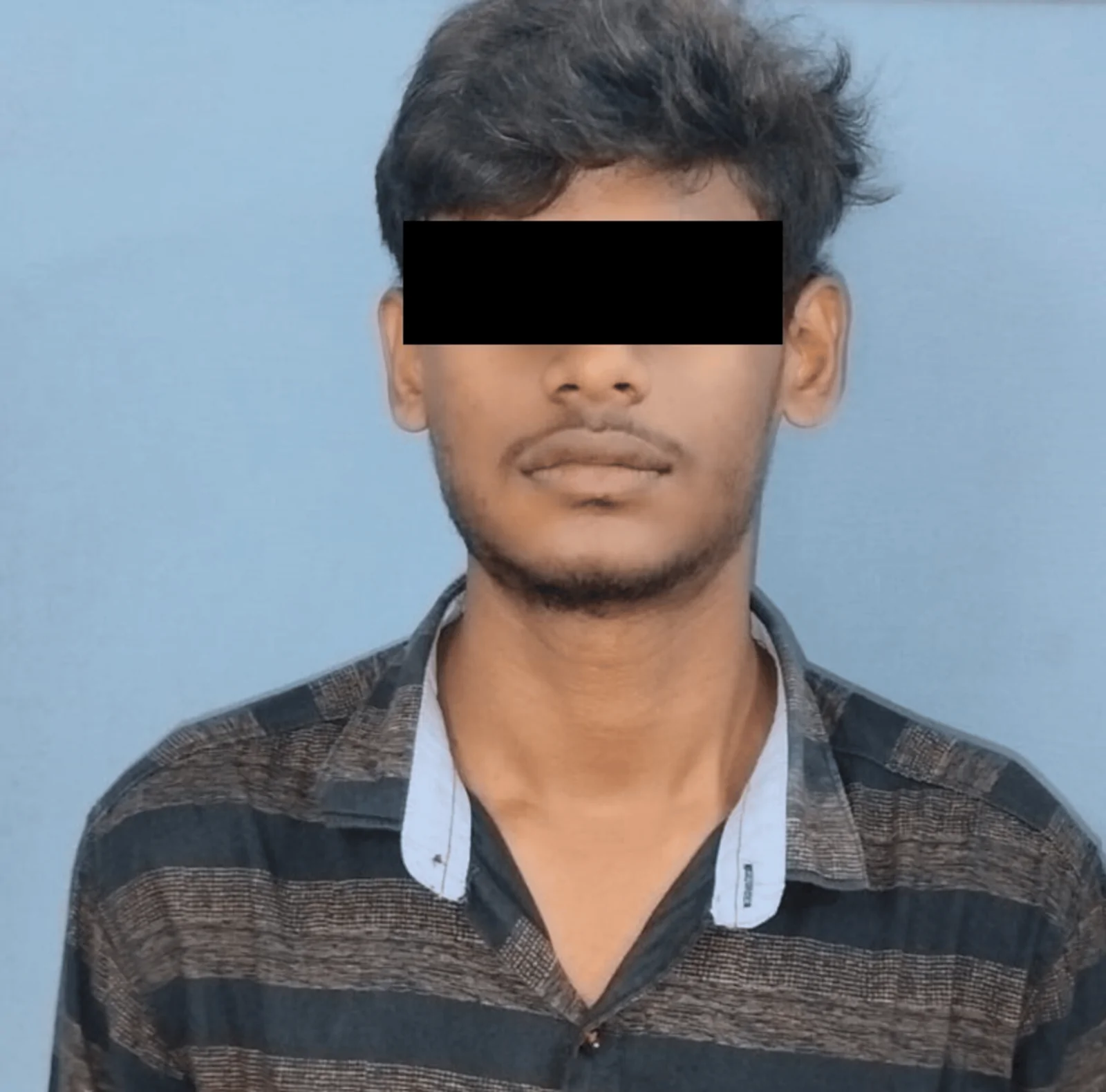
Extraoral clinical profile

A 21-year-old male reported to our department with a chief complaint of soreness and pain in his mouth for the past two days. The pain gets aggravated during the consumption of spicy, acidic foods (Figure 1).

**Figure 2:**
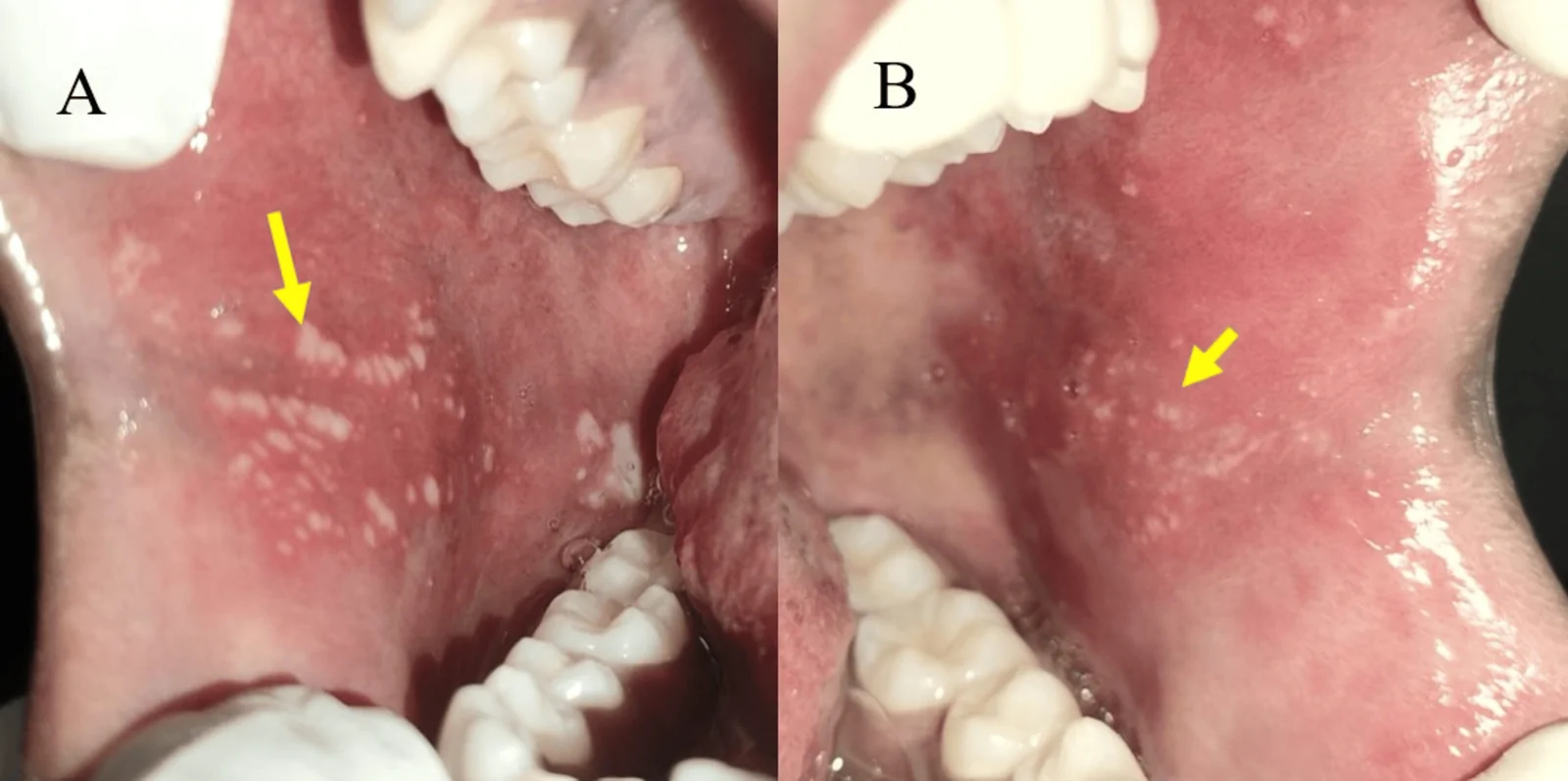
Multiple tiny, grouped ulcers around 35 in number measuring about 1 mm in diameter found both on the right (A) and left (B) buccal mucosa

A general examination revealed his vitals to be stable and afebrile, with no cervical lymphadenopathy. The patient had no similar ulcers in any other parts of his body or any skin lesions. The ulcers appeared the first time, and there was no history of recurrence of oral ulcers. The patient had no comorbidities such as hypertension, anemia, diabetes, or acquired immunodeficiency disease. The patient did not give any history of gastrointestinal upset, such as diarrhea. He was in emotional distress due to the sudden demise of his grandfather. Further history also revealed he hailed from a remote area where there is a lack of nutritious diet. The ulcers appeared for the first time insidiously after his emotional distress. Intraoral clinical examination revealed multiple discrete ulcers, each measuring about 1 mm in diameter and around 35 in number on both the left and right buccal mucosa and 45 tiny ulcers involving the right ventral surface of the tongue (Figure 2).

**Figure 3:**
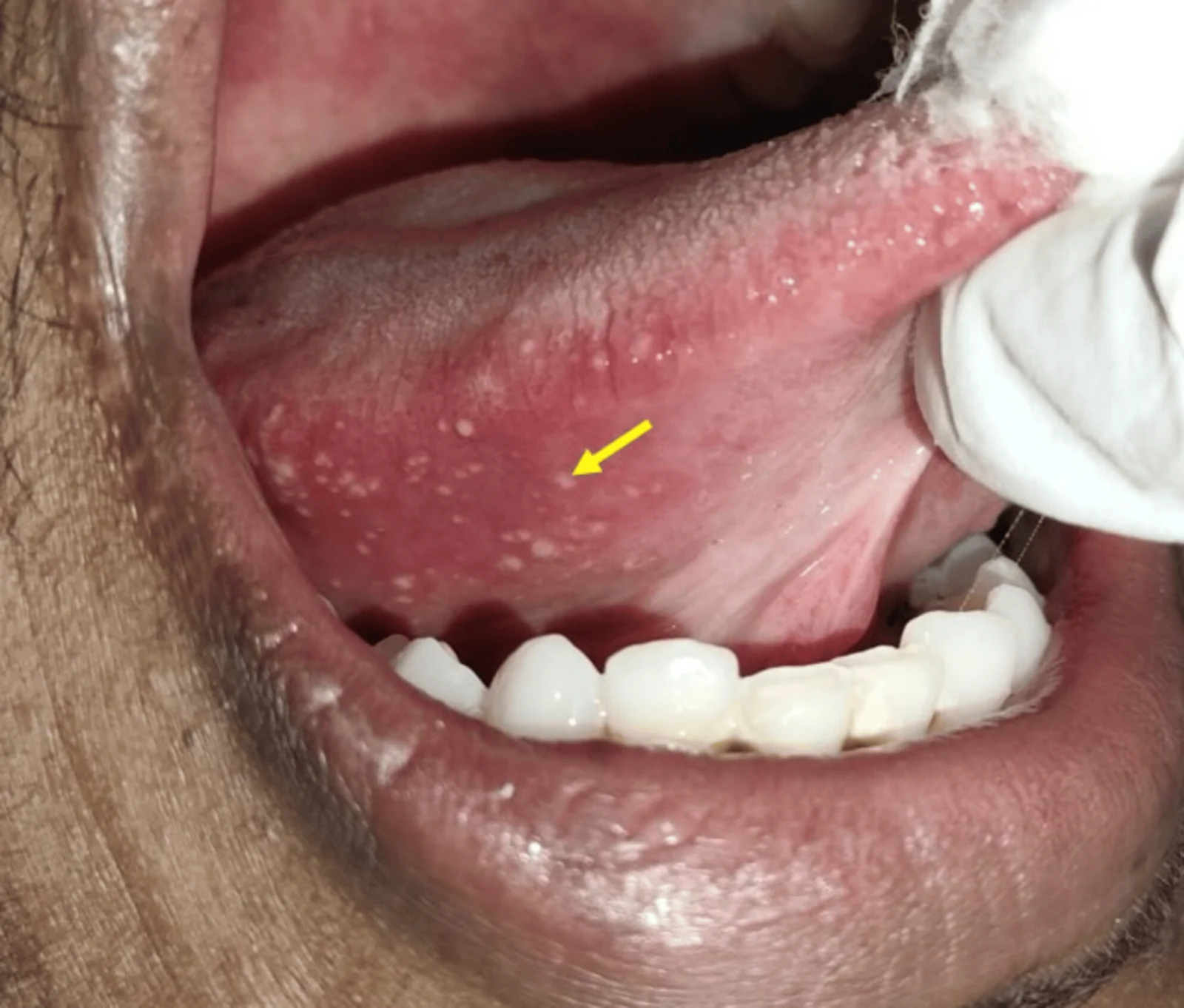
Clinical examination revealed multiple grouped tiny ulcers around 45 in number, each measuring 1 mm in diameter on the right-side ventral surface of the tongue

There was an absence of pasty or ropy saliva, and the patient had no metallic taste. Further inspection of the tongue revealed numerous grouped tiny ulcers, each 1 mm in diameter, on the right-side ventral surface of his tongue (Figure 3).

**Figure 4:**
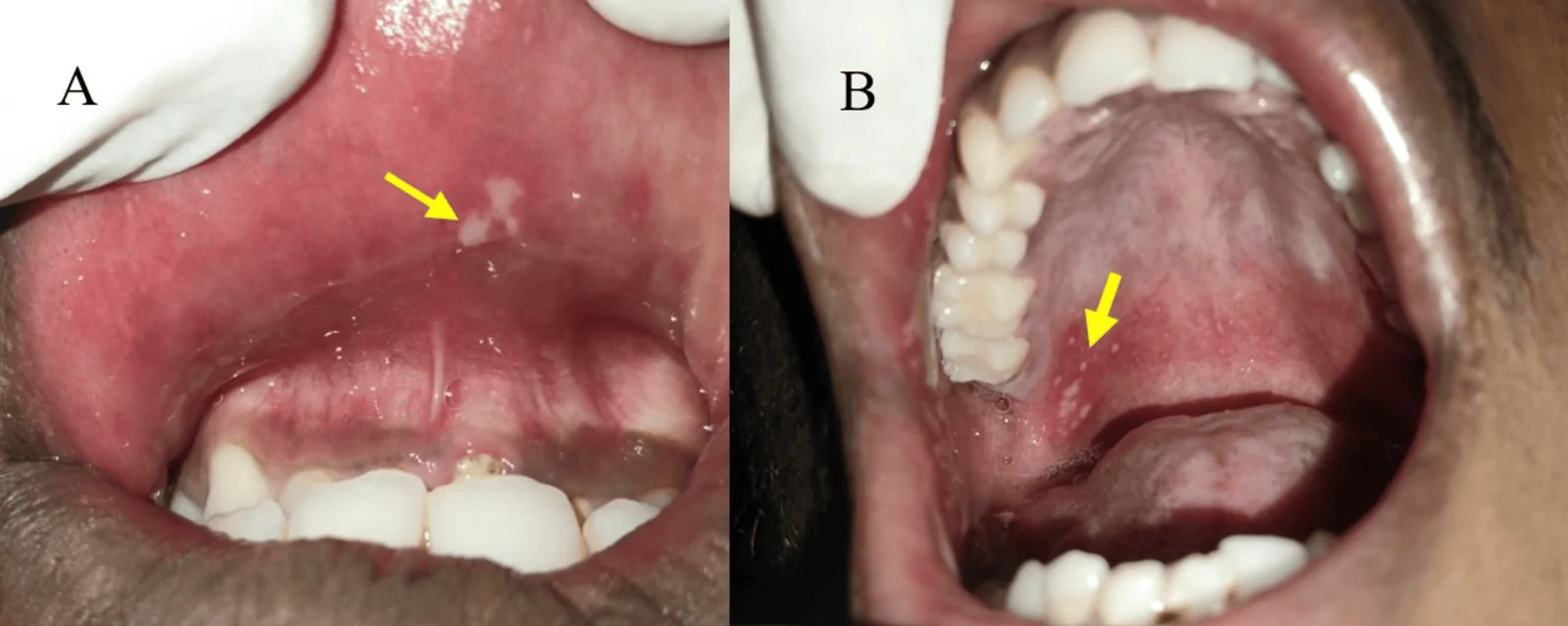
Multiple tiny ulcers of 1 mm in diameter on the maxillary labial mucosa and around 10 in number on the right posterolateral aspect (B) of the soft palate surrounded by linear erythematous area (A)

A careful closer clinical examination also revealed three discrete tiny ulcers that merged into a single ulcer involving the maxillary labial mucosa and around 10 tiny discrete ulcers, each of 1 mm in diameter, on the right posterolateral aspect of the soft palate (Figure 4).

The edges of the ulcer were sloping, and the floor showed a whitish slough. The surrounding areas of the ulcers revealed the presence of a linear erythema. On palpation, the base of the ulcers was non-indurated but tender. Based on the history, chief complaint, and intraoral clinical findings, a provisional diagnosis of HAU was made. The differential diagnoses were herpetic ulcers, cyclic neutropenia, and secukinumab-induced herpetiform oral ulcers. The hematological workup of the patient is shown in Table 1.

**Table 1:** Hematological parameters

Clinical parameters	Result	Normal reference range
Total white blood cell (WBC) Count	8500 Cells/Cumm	4000-11,000
Total red blood cells (RBC) Count	3.8 million Cells/Cumm	3.8-5.3 million Cells/Cumm
Mean corpuscular volume (MCV)	110 fl	80-100 fl
Mean corpuscular hemoglobin (MCH)	40.7 pg	27-32 pg
Mean corpuscular hemoglobin concentration (MCHC)	36.9 gm/dL	32-34 gm/dL
Red cell distribution width-coefficient of variation (RDW-CV)	18.6%	11.5 -14.5%
Red cell distribution width-standard deviation (RDW-SD)	73 fl	39-46 fl
Neutrophils %	69.5 %	50-70%
Lymphocytes %	19.2 %	11- 49 %
Hemoglobin	12.0 gm/dL	12-15 gm/dL
Platelet count	2,42,000 Cells/Cumm	1,50,000-5,00,000 Cells/Cumm

Unlike herpetic ulcers, HAUs are not caused by a viral infection. They are unique because they always occur insidiously without being preceded by multiple blisters on the mobile, non-keratinized mucosa, such as the tongue's ventral surface, the floor of the mouth, and the soft palate. In contrast, oral herpetic ulcers are caused by the contagious herpes simplex virus (HSV-1), and ulcers are preceded by the formation of tiny, multiple-grouped vesicles, which later ulcerate. Cyclic neutropenia was ruled out in our case as there was no fever, sore throat, cervical lymphadenopathy, or decrease in neutrophil count, and so were secukinumab-induced herpetiform aphthous-like oral ulcers in patients with psoriasis [7]. Our patient did not give any history of drug intake for skin lesions. Based on the history, clinical findings, and hematological parameters, a final diagnosis of megaloblastic anemia (based on the high mean corpuscular volume (MCV) and mean corpuscular hemoglobin concentration (MCHC) values) and stress-induced HAU were made.

**Figure 5:**
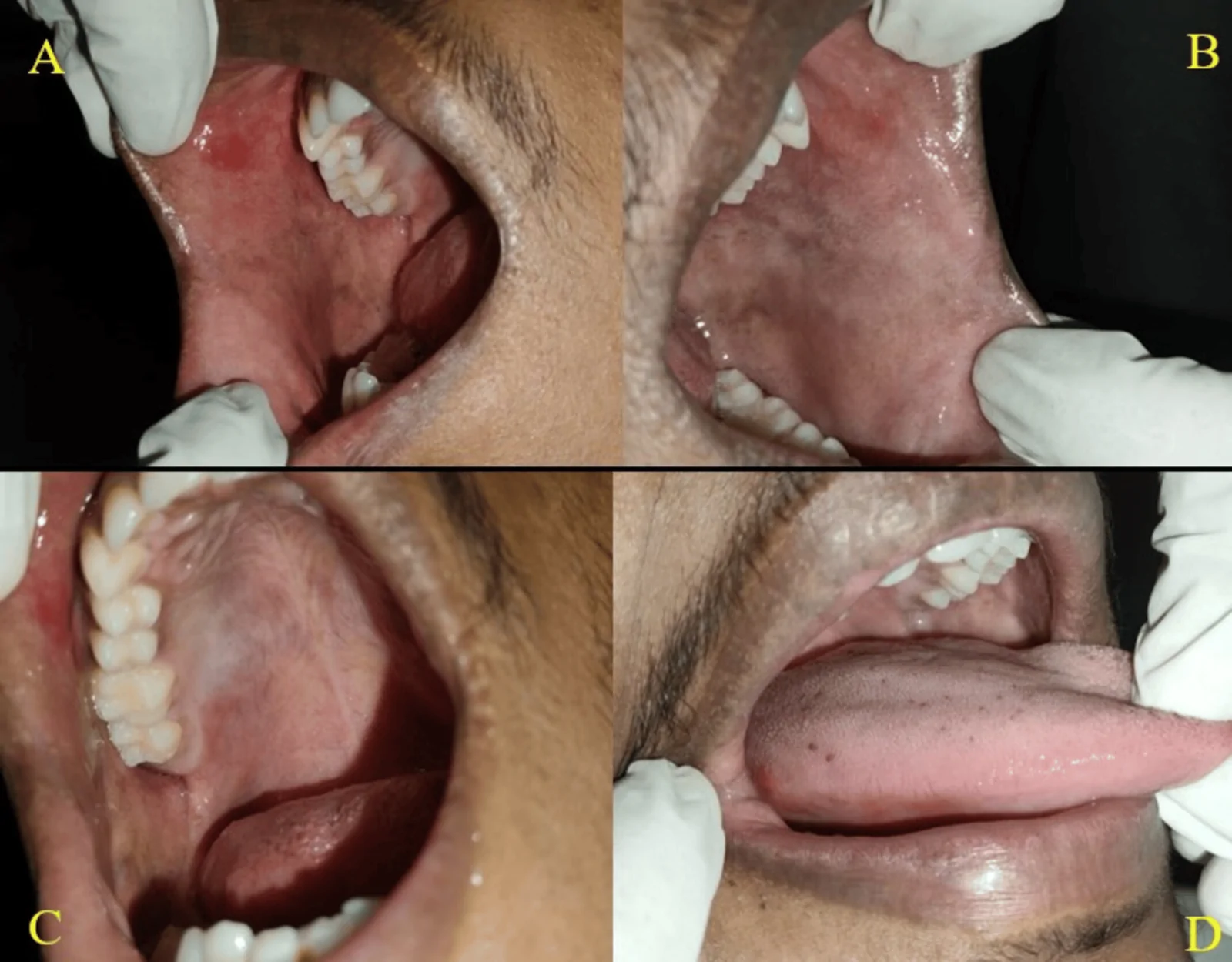
Follow-up Intraoral clinical photographs after two weeks revealed healed areas of the ulcers on the right buccal mucosa (A), left buccal mucosa (B), soft palate (C), and the floor of the mouth (D)

The patient was advised to dissolve 5 mg of tablet prednisolone in 150 ml of water, swish his oral cavity for five minutes once daily for two weeks, and spit. He was also advised to consume one capsule of vitamin B complex once daily for one month. Tablet venlafaxine 75 mg once daily in the morning was prescribed for two weeks to control his emotional distress. The follow-up intraoral clinical photographs were taken after two weeks (Figure 5). The clinical significance of this case report is the occurrence of a rare clinical form of aphthous ulcers, i.e., HAU, in a 21-year-old male patient amounting to about 126 multiple tiny oral ulcers.

## Discussion

Herpetiform aphthous ulcer is the least prevalent form of recurrent aphthous stomatitis (RAS), occurring in approximately 1% to 10% of patients with oral ulcers [1]. The terminology is derived from the clinical similarity of these lesions to primary herpetic stomatitis, without any association with herpes viruses. The HAU lesions clinically manifest as multiple, diminutive, abundant, and painful ulcers. They usually develop in clusters of up to 100 ulcers concurrently, measuring 2 mm to 3 mm in diameter, and persist for one to two weeks. The sites most commonly affected by HAU are the floor of the mouth and the tip and lateral edges of the tongue. These lesions clinically manifest on mobile, non-keratinized oral mucosa. Occasionally, these small-sized, tiny ulcers merge to form a larger and irregular ulcer, which heals with scarring [2,3]. Viral infections do not cause HAU and are contagious. The exact cause remains unclear, but multifactorial factors such as stress, trauma, and dietary sensitivities play a significant role in triggering outbreaks of HAU [2-4].

Characteristics of herpetiform ulcers

Herpetiform ulcers are typically very small, measuring about 1 mm to 2 mm in diameter, from one to 100 in number. They often appear as multiple pinpoint lesions that can cluster together, forming larger, irregularly shaped ulcers. This clustering can lead to significant areas of ulceration in the oral cavity. They have no gender predilection and are more common during the second decade of life [5]. Herpetiform aphthous ulcers can affect both keratinized and non-keratinized mucosal surfaces, including the ventral surface of the tongue, soft palate, and labial and buccal mucosa. The healing of HAU usually occurs within seven to 10 days without scarring but is extremely painful during this period [6]. The treatment of HAU focuses on symptom relief, such as the alleviation of pain, formation of a protective barrier, promotion of healing, and prevention of recurrence by the antioxidant mechanism by decreasing reactive oxygen species and immunomodulatory activity by suppression of T-lymphocytes, tumor necrosis factor-alpha activity, increasing interleukin (IL)-10, and epithelial growth factor (EGF) activity [7].

Treatment modalities 

Topical Medications

Topical anesthetics: The 5% lidocaine or 20% benzocaine gel, provides immediate pain relief by causing transient numbness of the affected ulcerative area [8].

Topical corticosteroids: The triamcinolone acetonide (Kenalog in Orabase) paste (1 mg/gm) applied twice or thrice daily creates a protective barrier over the ulcer, shielding it from additional irritation and harm. Thus, it facilitates an environment conducive to an expedited recovery [9].

Natural therapies: Curcumin, pomegranate (*Punica granatum*), honey (Propolis), and aloe vera aid in the healing of aphthous ulcers by the promotion of anti-inflammatory and antibacterial activity [10].

Systemic Medications

Corticosteroids: In cases of severe HAU, a short course of systemic steroids like prednisone is beneficial. However, it's important to note that long-term use of systemic corticosteroids is generally avoided due to potential side effects such as exacerbation of diabetes and peptic ulcers [10].

Immunomodulatory drugs: Immunomodulatory medications such as 0.5 mg and 2 mg colchicine are considered in chronic cases of aphthous stomatitis that do not respond to other treatments. Rebamipide 100 mg once daily and levamisole 50 mg once daily have proven beneficial. These medications work by modulating the immune system's response, which can be a contributing factor to the development of HAU [10].

## Conclusions

Herpetiform aphthous ulcers are a rare clinical variant of recurrent oral aphthous ulcers. The diagnosis of such HAU remains quite challenging due to their clinical resemblance to ulcers caused by herpes viral infections. Proper history taking, careful clinical examination, and hematological workup are essential for the diagnosis of HAU lesions. Herpetiform aphthous ulcers are often associated with significant discomfort due to their pain and multiple, grouped occurrences and severely impact the quality of life in affected individuals. Treatment of HAU is primarily symptomatic and focuses on pain relief, reduced inflammation, and promotion of healing.
